# Structural and functional analysis of a plant nucleolar RNA chaperone-like protein

**DOI:** 10.1038/s41598-023-36426-4

**Published:** 2023-06-14

**Authors:** Rita Fernandes, Anna Ostendorp, Steffen Ostendorp, Judith Mehrmann, Sven Falke, Melissa Ann Graewert, Magdalena Weingartner, Julia Kehr, Stefan Hoth

**Affiliations:** 1 Molecular Plant Physiology, Institute of Plant Science and Microbiology, Department of Biology, Universität Hamburg, Hamburg, Germany; 2 Molecular Plant Genetics, Institute of Plant Science and Microbiology, Department of Biology, Universität Hamburg, Hamburg, Germany; 3 Center for Free-Electron Laser Science (CFEL), Deutsches Elektronen Synchrotron (DESY), Notkestrasse 85, 22607 Hamburg, Germany; 4 European Molecular Biology Laboratory (EMBL) Hamburg Unit, Hamburg, Germany

**Keywords:** Structural biology, SAXS, Plant sciences, Plant molecular biology

## Abstract

Ribosome biogenesis is a key process in all eukaryotic cells that requires hundreds of ribosome biogenesis factors (RBFs), which are essential to build the mature ribosomes consisting of proteins and rRNAs. The processing of the required rRNAs has been studied extensively in yeast and mammals, but in plants much is still unknown. In this study, we focused on a RBF from *A. thaliana* that we named NUCLEOLAR RNA CHAPERONE-LIKE 1 (NURC1). NURC1 was localized in the nucleolus of plant cell nuclei, and other plant RBF candidates shared the same localization. SEC-SAXS experiments revealed that NURC1 has an elongated and flexible structure. In addition, SEC-MALLS experiments confirmed that NURC1 was present in its monomeric form with a molecular weight of around 28 kDa. RNA binding was assessed by performing microscale thermophoresis with the Arabidopsis internal transcribed spacer 2 (ITS2) of the polycistronic pre-rRNA precursor, which contains the 5.8S, 18S, and 25S rRNA. NURC1 showed binding activity to the ITS2 with a dissociation constant of 228 nM and exhibited RNA chaperone-like activity. Our data suggested that NURC1 may have a function in pre-rRNA processing and thus ribosome biogenesis.

## Introduction

During development and in response to different stimuli from the environment, plants are capable to adapt their proteome by regulating the balance between protein degradation and synthesis. The latter depends on ribosome biogenesis that requires the synthesis, processing, and assembly of four different ribosomal RNAs (rRNAs) and about 80 ribosomal proteins (RPs). This protein synthesis machinery for translation is conserved in evolution. As other eukaryotes, plants carry cytosolic 80S ribosomes that consist of a large 60S ribosome subunit (LSU) carrying 5S, 5.8S, and 25S ribosomal RNAs as well as 40 to 48 RPs and of a small 40S ribosome subunit (SSU) carrying the 18S rRNA and about 30 RPs^1,2^. While ribosomes have for a long time been regarded as static multi-protein complexes, there is increasing evidence for ribosome heterogeneity that may be regarded as an additional layer for the regulation of translation. On the one hand, studies on changes of the translatome in plants have indicated the regulation of translation in development and by external stimuli, including light conditions, hypoxia, and heat stress^3^. On the other hand, the concept of ribosome heterogeneity is supported by the tissue- or stress-dependent regulation of plant genes encoding RPs that has been shown by transcriptomics approaches^4,5^. It is still an open question whether changes in RP abundance indeed result in the biogenesis of heterogenous ribosomes and thus translational specificity in plants.

Whereas the final maturation of ribosomes takes place in the cytoplasm, early steps of ribosome biogenesis start in the nucleus. Among these steps are the transcription of rDNAs into pre-rRNAs and the processing of pre-rRNAs into mature rRNAs. While the 5S rRNA transcript is generated and processed in the nucleoplasm^6^, the polycistronic pre-rRNA precursor, which contains the 5.8S, 18S, and 25S rRNA, is processed in the nucleolus. The pre-rRNA precursor carries two internal transcribed spacers (ITS1 and ITS2) between the mature rRNAs and external transcribed spacers (5’-ETS and 3’-ETS) at both ends. Processing of the pre-rRNA that requires the catalytic activity of exo- and endonucleases to cleave and remove the transcribed spacers can vary between organisms^7^. In plants, two distinct rRNA biogenesis pathways have been shown depending on the timing of nucleolytic cleavage events^8–10^. The 5’-ETS-first pathway that starts with the removal of the 5’-ETS followed by ITS1 cleavage resembles the yeast rRNA biogenesis pathway. However, the major contribution to rRNA biogenesis has been attributed to the ITS1-first pathway that is also found in humans^11–13^. The ITS1-first pathway starts with cleavage at the ITS1 followed by 5’-ETS removal. Recently, the existence of a third pathway, ITS2-first, has been suggested that may be specific to plants^14^. Ultimately, all three different pathways lead to the formation of mature 5.8S, 18S, and 25S rRNAs.

The processing of the polycistronic pre-rRNA can be mediated by the 90S/ SSU processome and by pre-60S particles, which both contain various ribosome biogenesis factors (RBFs)^1,2^. Proteome analysis revealed 319 putative nucleolar proteinaceous RBFs in *Arabidopsis thaliana*^15,16^. In yeast, rapid affinity purification using a tagged version of the pre-rRNA processing factor Nop15, which is associated with 90S and pre-60S particles^17^, allowed for the actual identification of components of the respective complexes with almost no contamination^18^. The overlap between RBFs from yeast and Arabidopsis indicated that both, 90S and pre-60S particles may co-exist also in plants^2^. Several Arabidopsis orthologs of protein components of the Nop15 interactome have been identified and characterized. For example, nucleolar-localized STRESS RESPONSE SUPPRESSOR 1 and 2 are orthologs of yeast Has1 and were involved in heat stress responses^19^. Participation in pre-rRNA processing has been shown for the Arabidopsis orthologs of yeast Nob1, Noc4, and Enp1^20^. The Arabidopsis nucleostemin NSN1 (ortholog to yeast Nug1) is required for maturation of the pre-60S subunit and has been shown to interact with EBP2 (Epstein-Barr virus nuclear antigen binding protein2, ortholog to yeast Ebp2)^21,22^. The Arabidopsis orthologs BRX1-1 and BRX1-2 of the yeast protein Brx1 have been shown to have redundant functions in pre-rRNA processing^12^. An ortholog of the yeast Nop15 protein itself has not been studied in plants yet.

In yeast, the Nop15 protein binds to the ITS2, and depletion of Nop15 resulted in cell growth arrest and inhibition of 25S and 5.8S rRNA synthesis^17,23^. It has been shown by cryo-electron microscopy that Nop15 assembles with additional factors around a structure extending from the 5’-end of the 25S rRNA and the 3’-end of the 5.8S rRNA, probably binding directly to ITS2^24,25^. The crystal structure of Nop15 resolved the predicted RNA recognition motif (RRM) that contains two RNA-binding motifs (RNP1 and RNP2) and a highly flexible C-terminus that is also important for RNA binding^26^. Processing at the ITS2 depends on dynamic conformational changes between its ring and its hairpin structure^27^. It was shown by in vivo chemical probing that the transition from the ring to the hairpin structure may be regulated by the binding of Nop15 and its binding partner Cic1^23,28^. Depletion of Nop15 and Cic1 favored the hairpin structure that is potentially more stable^23^. In plants, the processing of the polycistronic pre-rRNA at the ITS2 has not been studied in detail. Therefore, we aimed to identify the structure and function of the Arabidopsis Nop15 ortholog and its capacity to bind to ITS2.

## Materials and methods

### Cloning of DNA constructs and plant transformation

For subcellular localization studies, the full-length coding sequence (CDS) of At5g04600, At5g05210, and At3g22660 excluding the STOP codon was amplified by PCR using the primers listed in Supplementary Table S1 and cloned into pDONR221. To obtain C-terminal GFP fusion constructs, this cloning was followed by the LR recombination reaction with the destination vector pABindGFP^29^. In the same way, the CDS of *MAIL1* was cloned into pABindmCherry^29^. For transient expression the constructs were transformed into *Agrobacterium tumefaciens* strain C58C1. The plasmid-containing agrobacteria were cultivated overnight at 28 °C, harvested by centrifugation, and the pellet was resuspended in infiltration buffer (10 mM MgCl_2,_ 10 mM 2-(N-morpholino)ethanesulfonic acid, 200 µM acetosyringone) to a final OD600 of 0.6. The agrobacterium suspension was infiltrated into leaves of 4- to 6-week-old *Nicotiana benthamiana* plants using a needleless 2-mL syringe. The GFP fluorescence was analyzed three days after infiltration. All plant experiments were performed in accordance with the relevant regulations and legislations.

For the expression of NURC1 in *E. coli* total RNA was extracted from *A. thaliana* leaves and, subsequently, reverse transcribed by RT-PCR with the RevertAid First Strand cDNA synthesis kit (ThermoFisher Scientific). The DNA corresponding to the full length *NURC1* coding sequence (AT5g04600) was amplified with Phusion polymerase (Thermo Fisher Scientific), purified by NucleoSpin^®^ Gel and PCR Clean-up kit (Macherey–Nagel) and cloned into pET-28a(+) using NdeI and XhoI restriction sites. The truncated versions were achieved by adding a stop codon at positions 140 and 160 through site directed mutagenesis with Phusion polymerase (Thermo Fisher Scientific). Template plasmid was removed by performing *DpnI* digestion at 37 °C for 1 h. NURC1_53-222_ was amplified from pET28a(+)-NURC1 FL with primers that include *BsaI* recognition sites and was inserted into pET28a(+) by Golden Gate cloning with *BsaI*. The sequences were confirmed by Sanger sequencing (Microsynth). All primer sequences are listed in Supplementary Table S1.

### Confocal microscopy

To detect fluorescence of GFP or mCherry, confocal laser scanning microscopy was applied by using the Leica TCS SP8 Confocal Platform (Leica Microsystems, Wetzlar, Germany). For excitation of GFP and mCherry laser light of 488 nm and 561 nm was used, respectively. The detection windows ranged from 496 to 511 nm (GFP) and 569–591 nm (mCherry).

### Expression and purification of NURC1 proteins

The expression was carried out at 24 °C overnight in the *E. coli* strain BL21-CodonPlus (DE3)-RIPL by autoinduction. The cells were harvested at 4 ºC and 5000 rpm for 30 min (JA-10 rotor, Avanti JXN-30 Beckman Coulter). The pellets were resuspended in lysis buffer (50 mM HNa_2_PO_4_ pH 7.5, 200 mM NaCl, 30 mM imidazole, 5% glycerol (v/v), 1 mM DTT, 1 mM PMSF). No reducing agent was used for NURC1_1-140_ purification. Protease inhibitor (1 pill, Roche), Ribonuclease A (q.s., Carl Roth) and DNaseI (q.s., PanReac AppliChem) were added to the resuspension mixture. Lysis was performed by adding 1 mg/ml of lysozyme and incubating at 4 °C, for 40 min with stirring, followed by sonication (8–10 times 30 s on + 30 s off, 45% duty cycle, output control 5, Branson sonifier 250). The cell debris was removed by centrifugation at 4 °C, 14,000 rpm for 30 min (JA-25.50 rotor, Avanti JXN-30 Beckman Coulter) and the supernatant filtered with a 45 μm filtropur S. Immobilized matrix assisted chromatography (IMAC) was conducted using a 5 ml HisTrap HP (GE Healthcare). The column was washed with 50 ml of washing buffer (50 mM HNa_2_PO_4_ pH 7.5, 1 M NaCl, 30 mM imidazole, 5% glycerol (v/v), 1 mM DTT) followed by elution with a linear gradient from 30 mM to 1 M of imidazole on an ÄKTA system (Äkta prime plus, GE Healthcare). Dialysis (50 mM HNa_2_PO_4_ pH 7.5, 200 mM NaCl, 5% glycerol (v/v), 1 mM DTT, 1 mM EDTA) and 6xHis-tag cleavage (1 U/ml final concentration of thrombin) were combined and performed overnight at 4 °C. The dialysed solution was centrifuged to remove any larger particles and concentrated. Further purification was achieved by size exclusion chromatography (SEC) using a HiLoad™ 16/600, Superdex™ 200 pg (GE Healthcare) column at 4 °C preequilibrated with running buffer (HNa_2_PO_4_ pH 7.5, 300 mM NaCl, 5% glycerol (v/v), 1 mM DTT). Fractions with highest purity were combined and concentrated. The purity and identity of each protein were determined by SDS-PAGE and MALDI-TOF MS, respectively.

### In vitro transcription and RNA labelling

Genomic DNA from *A. thaliana* leaves was extracted and the 45S rDNA amplified, gel extracted and purified by NucleoSpin^®^ Gel and PCR Clean-up kit (Macherey–Nagel). The PCR fragment was cloned into pUC57 by the Gibson Assembly cloning method. The ITS2 region was checked by Sanger sequencing (Microsynth) using designed primers. Using the construct as template, full length ITS2 (containing T7 promotor sequence) was amplified by touchdown PCR with Phusion polymerase (Thermo Fisher Scientific), followed by NucleoSpin^®^ Gel and PCR Clean-up kit (Macherey–Nagel). In vitro transcription of ITS2 was adapted from a published protocol^30^. In house purified T7 RNA polymerase was used. ITS2 FL was obtained by mixing 10 pmol of the DNA template in transcription buffer (50 mM Tris–HCl pH 7.5, 15 mM MgCl_2_, 5 mM DTT and 2 mM spermidine) with 0.1 mg/ml of T7 RNA polymerase, 2 mM of ATP, 2 mM of GTP, 2 mM CTP, 0.005 U/µl of inorganic phosphatase, 10% (v/v) DMSO and 1 U/µl of RiboLock RNase Inhibitor (ThermoFisher Scientific). A 1:15 (cy5-UTP/UTP) ratio of final molar concentration was used. The mixture was incubated for 2 h at 37 °C and covered from light. After synthesis, the DNA template was removed by DNase I (ThermoFisher Scientific) digestion. The final RNA product was purified via RNA Clean & Concentrator kit (Zymo Research). RNA concentration and quality were determined via nanodrop (Nanodrop one C, Thermo Scientific) and bioanalyzer (Agilent 2100 bioanalyzer). ITS2 fragments 1–79, 82–162 and 157–187 were synthesized following the same protocol. However, annealed primers were used as template instead. Primer annealing was done for 5 min at 95 °C. The primers sequences are listed in Supplementary Table S1.

### Microscale thermophoresis (MST)

The binding affinities between protein and RNA were determined by MST. The concentration of fluorescently labelled RNAs was kept fixed at 10 nM. The concentration of proteins was in the nano- to micromolar range. The protein/RNA mixture was spun down and kept at room temperature for 5 min in 20 mM Tris–HCl pH 8.0, 150 mM NaCl, 0.1 mg/ml BSA, 1 mM DTT and 0.1% Tween-20 (v/v). The samples were loaded into Monolith NT.115 capillaries and measured in a Nanotemper Monolith NT.115 device (100% red LED and medium MST power). Data analysis was done using the MO.Affinity analysis software. The SD-test was performed by centrifuging the mixture of protein and RNA for 10 min at maximum speed, using low binding tubes. For each sample, 15 µl were carefully removed and mixed with 15 µl of SD-mix (4% SDS, 40 mM DTT) in a new tube. All samples were incubated for 5 min at 95 °C. The tubes were spun down before loading the samples into the capillaries.

### RNA chaperone-like activity assay

The RNA chaperone activity assay was based on a previously published protocol^31^, with some minor changes. The same complementary 21-nucleotide oligoribonucleotides were used (21R + and 21R-). RNA annealing activity was tested by mixing 10 nM of 21R + strand with 50 nM of protein and incubating at room temperature for 1 min in 50 mM HEPES pH 7.5, 3 mM MgCl_2_ and 1 mM DTT. Hereafter, 10 nM of 21R- strand was added to the previous mixture and homogenized by pipetting up and down. The fluorescence measurements were recorded at 37 °C in a 96-well flat black plate with a Spark^®^ multimode microplate reader (Tecan Austria GmbH). The curve fitting was done in OriginPro 2021b (OriginLab) to an exponential one-phase association model.

### Dynamic light scattering (DLS)

DLS measurements were performed in a SpectroLight 600 Instrument (Xtal Concepts) using a Terazaki 72-well plate covered with paraffin oil to expose the sample solution drops. The instrument contains a red-light diode laser (660 nm) and detects the scattered light at a scattering angle of 142° using a photomultiplier tube. Protein solutions were centrifuged at maximum speed (20,000 × g) for 10 min at 4 °C. The protein concentration was determined by absorbance at 280 nm using a nanodrop 2000c UV/vis spectrophotometer (Thermo Scientific). All samples were kept at 4 °C until measured. Scattering measurements in 5 µl drops were taken 10 to 20 times for 10 s at 20 °C and averaged. The hydrodynamic radii distribution was calculated based on the diffusion constants using the Stokes–Einstein equation.

### Small angle x-ray scattering (SAXS)

Both static SAXS (batch mode) as well as SAXS coupled to SEC (SEC-SAXS) were performed at the synchrotron beamline P12 operated by EMBL Hamburg at the PETRA III (DESY, Hamburg, Germany). General beamline configuration parameters as well as SEC and sample parameters are listed in Supplementary Table S2. The data were normalized to the intensity of the transmitted beam and radially averaged. The scattering of the solvent-blank was subtracted. In batch mode this was achieved through a separate measurement of solely the buffer, in SEC mode frames before and after the elution peak were averaged and used for background subtraction. Final scattering curves are presented as I(s) vs s, where s = 4πsinθ/λ, and 2θ is the scattering angle. For batch mode 3 single measurements at different solute concentrations (10.5, 2.8, 1.44 mg/ml) were measured at 20 °C. Here, 20 successive 0.045 s frames were collected and averaged.

For SEC-SAXS, the protein sample was centrifuged at 20,000× g for 10 min at 4 °C. A Superdex^®^ 75 Increase 10/300 GL (GE Healthcare) column was used with a flow rate of 0.5 ml/min and 90 µl of sample at 18 mg/ml was loaded. SEC running buffer as in the protein purification protocol was used as mobile phase. One second frames were measured continuously. Data processing and modelling were done using ATSAS 3.0 package tools^32^. Specifically, the program Crysol was used for assessment of the fit of various models. GASBOR and DAMMIN were used for ab initio modelling and SREFLEX for normal mode analysis.

### Size exclusion chromatography- multi angle laser light scattering (SEC–MALLS)

The protein samples were centrifuged at 20,000 g for 10 min at 4 °C. A Superdex^®^ 75 Increase 10/300 GL (GE Healthcare) column was used with a flow rate of 0.5 ml/min. Loading concentration and amount information can be found in Supplementary Table S3. The running buffer was the same as in the SEC-SAXS experiment. MALLS measurements were performed at P12 beamline with an on-line UV–vis/MALLS/QELS/RI system. Scattering was recorded by a miniDAWN^®^ TREOS^®^ MALLS detector, with an in-built Quasi-Elastic Light Scattering (QELS) module and an Optilab T-rEX (RI) refractometer (Wyatt). The MALLS system was calibrated to the light scattering of toluene. Molecular weight estimate was done by taking three-angle MALLS scattering intensities combined with the protein concentration determined by RI using the ASTRA 7 software package (Wyatt Technology Corporation). Hydrodynamic radius (R_h_) was determined by the QELS module. Correction to the solvent viscosity was performed due to the presence of 5% (v/v) glycerol in the running buffer.

## Results

### Plant orthologs of yeast pre-60S components were localized to the nucleolus

**Figure 1:**
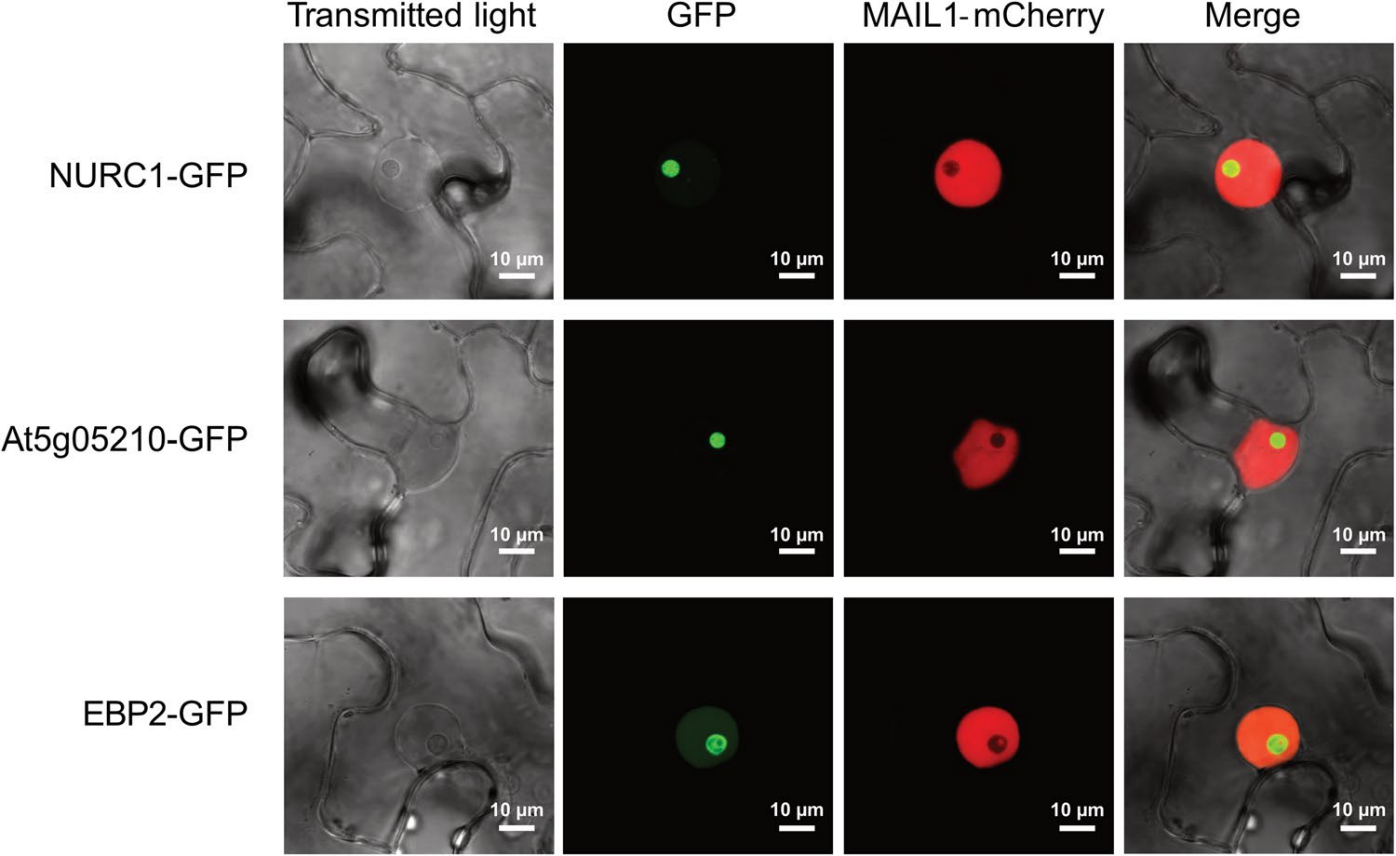
NURC1 and other plant RBFs are localized in the nucleolus. Subcellular localization of GFP-tagged NURC1, At5g05210 and EBP2 (At3g22660) transiently co-expressed with the nuclear marker MAIL1-mCherry in *N. benthamiana* leaf cells. Fluorescence of the respective fusion proteins was analyzed by using confocal microscopy. The merged pictures indicated the localization of all GFP fusion proteins in the nucleolus.

In a first step, we aimed to investigate whether the Arabidopsis orthologs of yeast Nop15 and other components of the nucleolar Nop15 pre-60S complex were also localized to the nucleolus in plant cells. To this end, we selected the Nop15 ortholog At5g04600 (hereafter named NUCLEOLAR RNA CHAPERONE-LIKE 1, NURC1), the uncharacterized Rrp14 ortholog At5g05210, and the Ebp2 ortholog At3g22660 (also named EBP2). The EBP2 protein has previously been shown to have a function for plant growth and to localize to the nucleolus in plant cells^21^. For all three RBFs we generated DNA constructs for the expression of the respective GFP fusion proteins in plant cells. After Agrobacteria-mediated transformation of *35S::NURC1-GFP*, *35S::At5g05210-GFP*, and *35S::EBP2-GFP* in *Nicotiana benthamiana* leaf epidermis cells, we analyzed the fluorescence of the GFP fusion proteins by confocal laser scanning microscopy (Fig. 1).

The co-expressed nuclear marker MAIL1 fused to mCherry was used to label the nuclei in transformed cells^33^. The GFP fluorescence signals in those mCherry-positive cells indicated the localization of all three fusion proteins, NURC1-GFP, At5g05210-GFP, and EBP2-GFP in nucleoli inside the respective nucleus (Fig. 1), suggesting a role of the proteins in the nucleolus. In the following, we studied structure and function of the Nop15 ortholog NURC1.

### NURC1 has an elongated shape and contains a RNA binding motif

**Figure 2:**
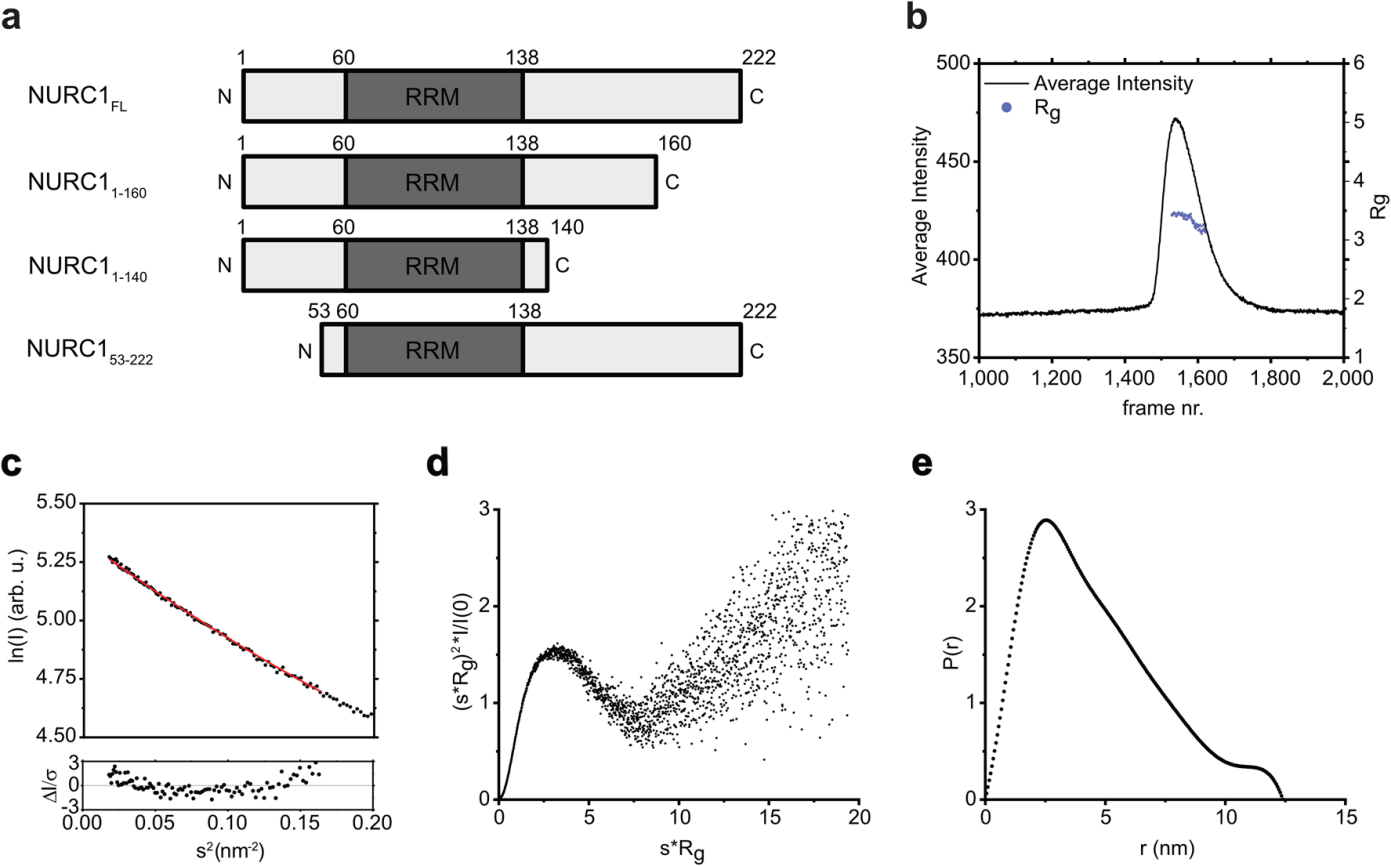
SEC-SAXS analysis of NURC1_FL_ and truncated NURC1 proteins. (**a**) Representation of NURC1_**FL**_ and NURC1 proteins with truncations at positions 160, 140 and 53. The putative RNA recognition motif (RRM) is represented as dark grey box. (**b**) SEC chromatogram showing the average intensity (black line) and the R_g_ predicted values for the analyzed range of frames (purple spheres) of the NURC1 full-length protein. The loaded sample concentration was 18 mg/ml. (**c**) Guinier fit (red line) of the experimental data (black spheres) on the top and standardized residuals on the bottom. (**d**) Dimensionless Krakty plot. (**e**) P(r) versus r profile.

Following the structure prediction of AlphaFold (alphafold.ebi.ac.uk)^34,35^, the NURC1 protein is flexible at both ends and contains a highly confident structural domain in the middle part of the protein with similarity to a RNA recognition motif (RRM, c.f. uniport.org). To get experimental proof for the predictions and to resolve the actual NURC1 structure, we expressed the 222 amino acid-long NURC1 full-length protein (NURC1-FL) in *E. coli* for purification of the recombinant protein. For yeast it has been shown that the C-terminal residues affect solubility and RNA binding properties of the Nop15 protein^26^. We therefore additionally generated three truncated NURC1 proteins with deletions at the C- or N-terminus, namely NURC1_1-160_, NURC1_1-140_, and NURC1_53-222_ (Fig. 2a and Supplementary Fig. S1).

To resolve the structure of NURC1 in solution, we performed small angle X-ray scattering (SAXS) experiments in the batch mode at three different concentrations (Supplementary Fig. S2a). Guinier analysis of the scattering data showed that the radius of gyration R_g_ increased non-linearly, thus indicating undesired concentration dependency (Supplementary Fig. S2b). Dynamic light scattering (DLS) measurements supported and confirmed this concentration dependency of the hydrodynamic radius (R_h_) and indicated varying sample polydispersity (Supplementary Fig. S3a). Further, DLS of NURC1_1-160_, NURC1_1-140_ and NURC1_53-222_ samples showed that the truncations at the C-terminus induced polydispersity, and that the truncation at the N-terminus induced substantial aggregation (Supplementary Fig. S3b). To overcome the issue of concentration dependency, we performed SAXS measurements in line with size exclusion chromatography (SEC) that yielded one peak only (Fig. 2b). The Guinier approximation from the SEC-SAXS data resulted in a radius of gyration R_g_ of 3.52 ± 0.01 nm with sR_g_ limits between 0.47 and 1.29 (Fig. 2c). It is noteworthy to mention that the fitted low s values were linear (Pearson’s R value of − 0.998) and that the double logarithmic scale scattering curve had a null slope at low s values as well. These results sustained that similar sized and monodispersed particles were present in solution. SEC-MALLS was performed in addition to confirm size and oligomeric state of the sample. One large elution peak was observed with a small shoulder at a higher elution volume (Supplementary Fig. S4a). The obtained molecular weight was around 28 kDa (Supplementary Fig. S4b, Table S3). The closer value to the real molecular weight supports the idea of a monomeric state. Furthermore, from the QLES detection a hydrodynamic radius of 2.4 nm was obtained (Supplementary Fig. S4c, Table S3). The R_g_/R_h_ ratio is around 0.77 for globular proteins. NURC1 showed a ratio of 1.5 which agrees with an elongated shape. Moreover, we performed SEC-MALLS of NURC1_1-160_, NURC1_1-140_ and NURC1_53-222_ (Supplementary Fig. S4). In general, the SEC yielded one elution peak for each sample (Supplementary Fig. S4a). However, the NURC1_1-140_ elution peak was flanked by a shoulder at lower and higher elution volumes, respectively, suggesting that this truncated NURC1 protein may be more polydispersed when compared to the full-length NURC1.

A dimensionless Kratky plot was used to access conformational information (Fig. 2d). It is known that for globular proteins a bell-shaped peak is expected at s*R_g_= $\sqrt{3}$ ≈ 1.73 with a height of 1.1^36^. In the case of NURC1, the bell shape seemed to be in order, but it was clearly right-shifted (s*R_g_ = 3.26) and slightly higher [(s*R_g_^2^)*I/I(0) = 1.62], suggesting that NURC1 had an elongated shape and/ or was partially unfolded. Furthermore, values increased at s*R_g_ > 7.5 instead of converging with the axis, providing additional evidence for flexibility. A non-Gaussian curve with a peak at approximately 2.5 nm was obtained as the pair distance distribution function P(r) (Fig. 2e). A long tail that ended at a D_max_ of 12.4 nm and a small shoulder were visible, being again characteristic for an elongated macromolecule. Remarkably, the R_g_/I(0) ratios from the Guinier plot and P(r) function were in agreement, thus dismissing any presence of aggregates that have mostly an impact at lower s values, consequently in the Guinier approximation. The molecular weight can approximatly be determined by the Bayesian Inference. The monomeric form should be around 25 kDa, however the estimated molecular weight was around 33.8 kDa with a probability of 51.6%. Since the calculations for this estimation are fundamentally based on spherical shaped particles, it could be assumed that the size overestimation was most likely due to the elongated shape of NURC1.

In a next step , we were aiming to build a structure model for NURC1 based on the SEC-SAXS data. CRYSOL was used to test four different atomic models against the experimental scattering data (Supplementary Table S4). Two homology models consisted of atomic resolution structures (X-ray crystallography and CryoEM) and the other two were predicted models obtained by different tools. The AlphaFold model showed a χ^2^ of 11.97. Ab initio models were generated using DAMMIN and GASBOR. The DAMMIN P1 model showed χ^2^ of 1.06 and the GASBOR P1 model a χ^2^ of 1.56. The discrepancy between models and experimental data was not surprising due to the intrinsic disorder. A plethora of structural conformations can co-exist in solution and the resulting scattering is the average of them all, which renders the search for the correct structure model (in the right conformation) difficult. In this context, it should be noted that the resulting ab initio models do not necessarily represent an actual state of the protein itself but rather an ‘artificial’ averaged state.

**Figure 3:**
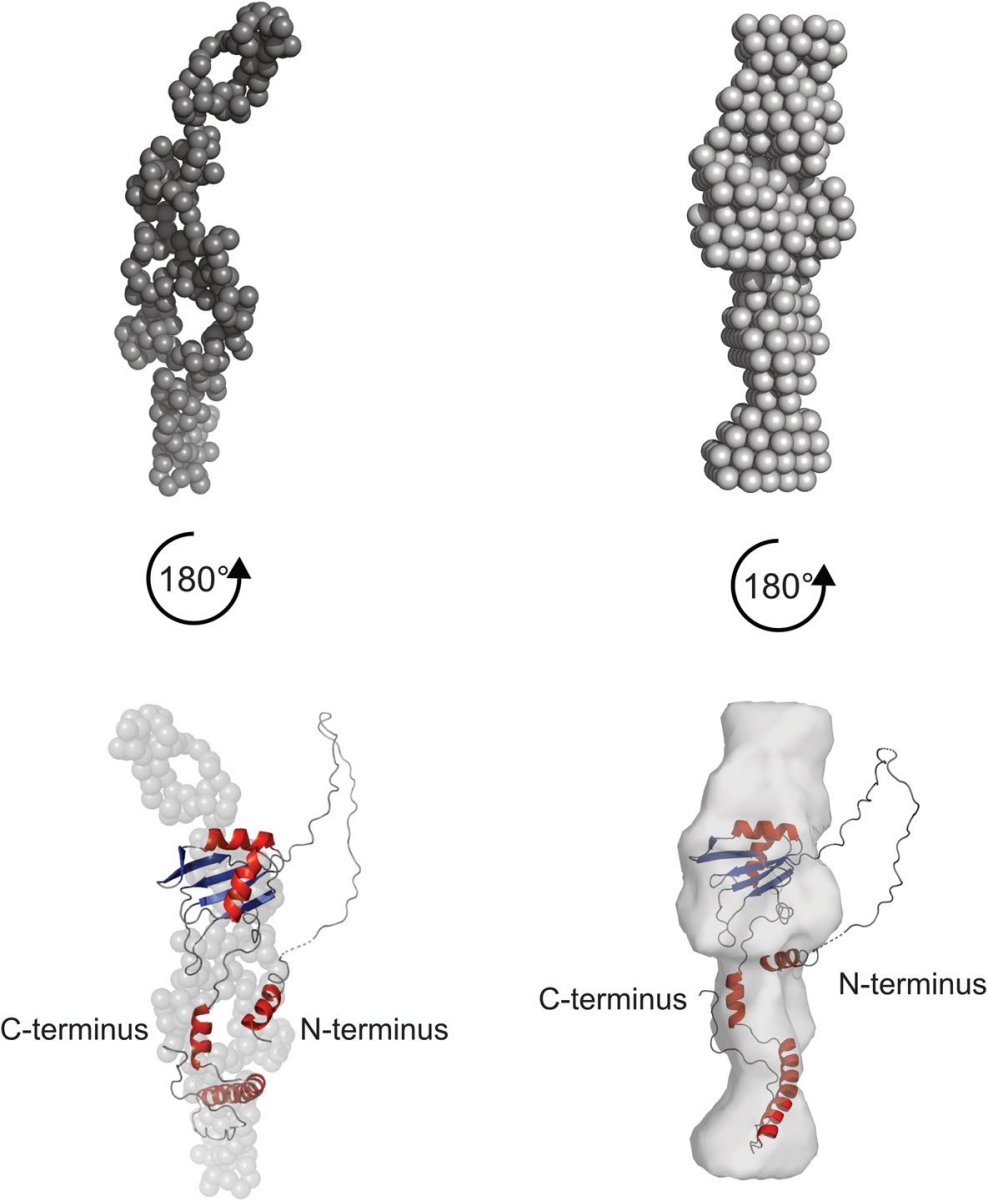
Superimposition of the GASBOR P1 and the DAMMIN P1 model with the SREFLEX model of NURC1_FL_. The GASBOR P1 model (top left, dark grey spheres), the DAMMIN model (top right, light grey spheres), and the respective superimpositions with the SREFLEX model (bottom left and right) are shown. The superimposition was achieved by running the supalm tool from the SASpy ATSAS plugin in Pymol. The superimposition with the GASBOR P1 and the DAMMIN model resulted in a normalized space discrepancy (NSD) of 4.77 and 5.36, respectively.

SREFLEX estimates the flexibility of pre-existing high-resolution models creating new models that aid in finding a conformation that fits the data better. The AlphaFold model was used as input. The resulting fit showed a major improvement (χ^2^ = 3.04). The DAMMIN P1 and GASBOR P1 models were superimposed with the SREFLEX top model (Fig. 3). The superimposition indicated that both termini did not fit perfectly. This was even more prominent for the N-terminus that besides being flexible was also highly disordered.

**Figure 4:**
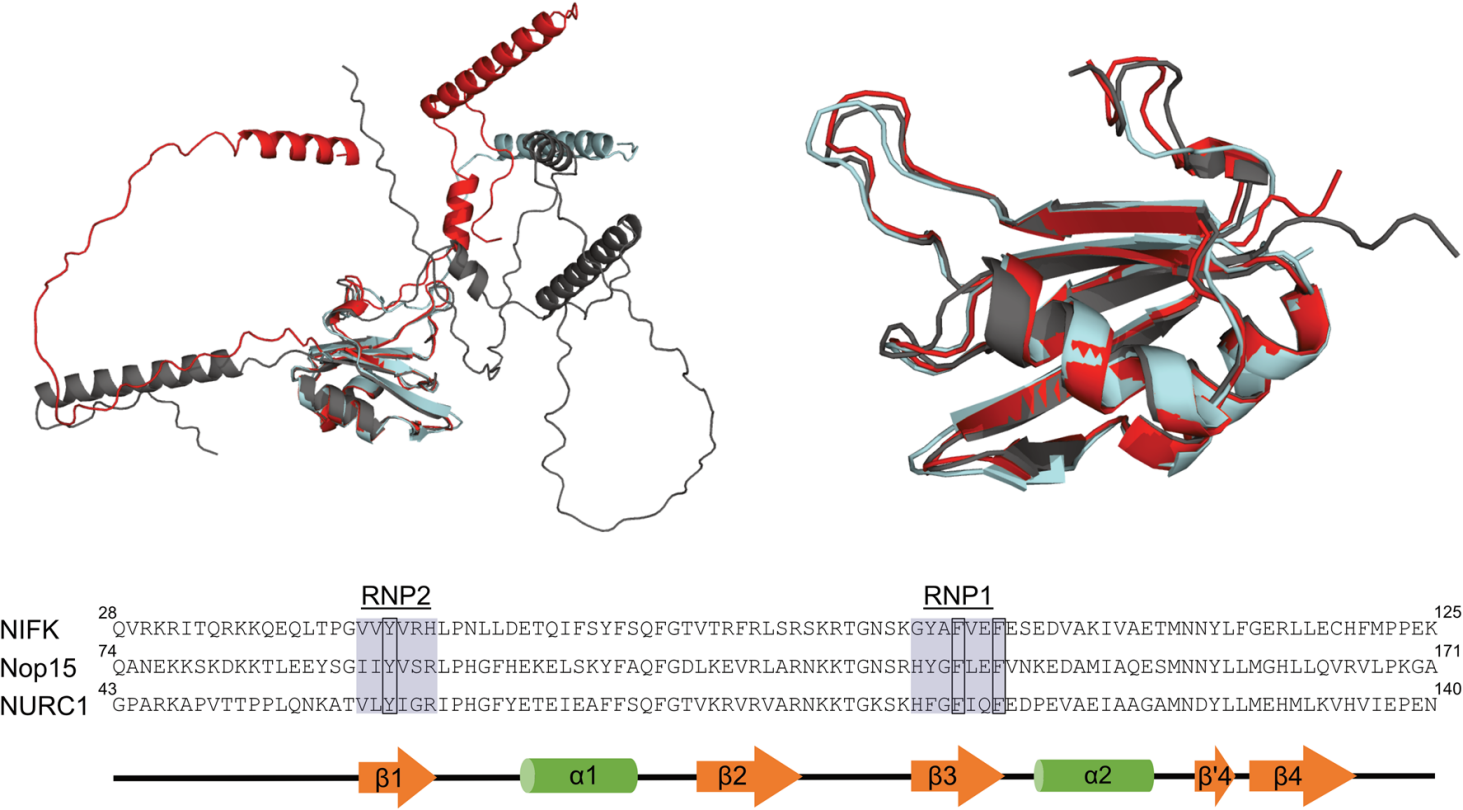
The RRM structure and RNPs aromatic residues are highly conserved. NURC1 AlphaFold model Q9LZ65 (red), NIFK AlphaFold model Q9BYG3 (grey) and Nop15 CryoEM structure PDB 3JCT (light blue) represented as cartoons (top left) and RRMs (top right). The superimposition of the three models was performed in Pymol by using the alignment plugin (align, outlier rejection, 20 cycles and a cutoff of 2). The Nop15 structure was used as target. The final RMSD values for NURC1 and NIFK were 0.595 and 0.776, respectively. Sequence alignment was performed using Clustal Omega for all three proteins (bottom). RNPs are highlighted in sky blue and conserved amino acids are inside boxes. The RRM secondary structure is displayed on the bottom as a cartoon where β-sheets are represented as orange arrows and α-helices as green rods.

The structure alignment of the NURC1 AlphaFold model, the Nop15 Cryo-EM structure and NIFK AlphaFold model indicated that all three RRMs superimpose to a high degree, with root mean square deviation (RMSD) values below one (Fig. 4). The sequence alignment showed that the aromatic residues F105 and F108 in RNP1 and Y63 in RNP2 are conserved in NURC1 (Fig. 4). This suggested that NURC1 may bind to RNA like its yeast ortholog Nop15.

### NURC1 binds to ITS2 RNA and binding depends on its C-terminus

**Figure 5:**
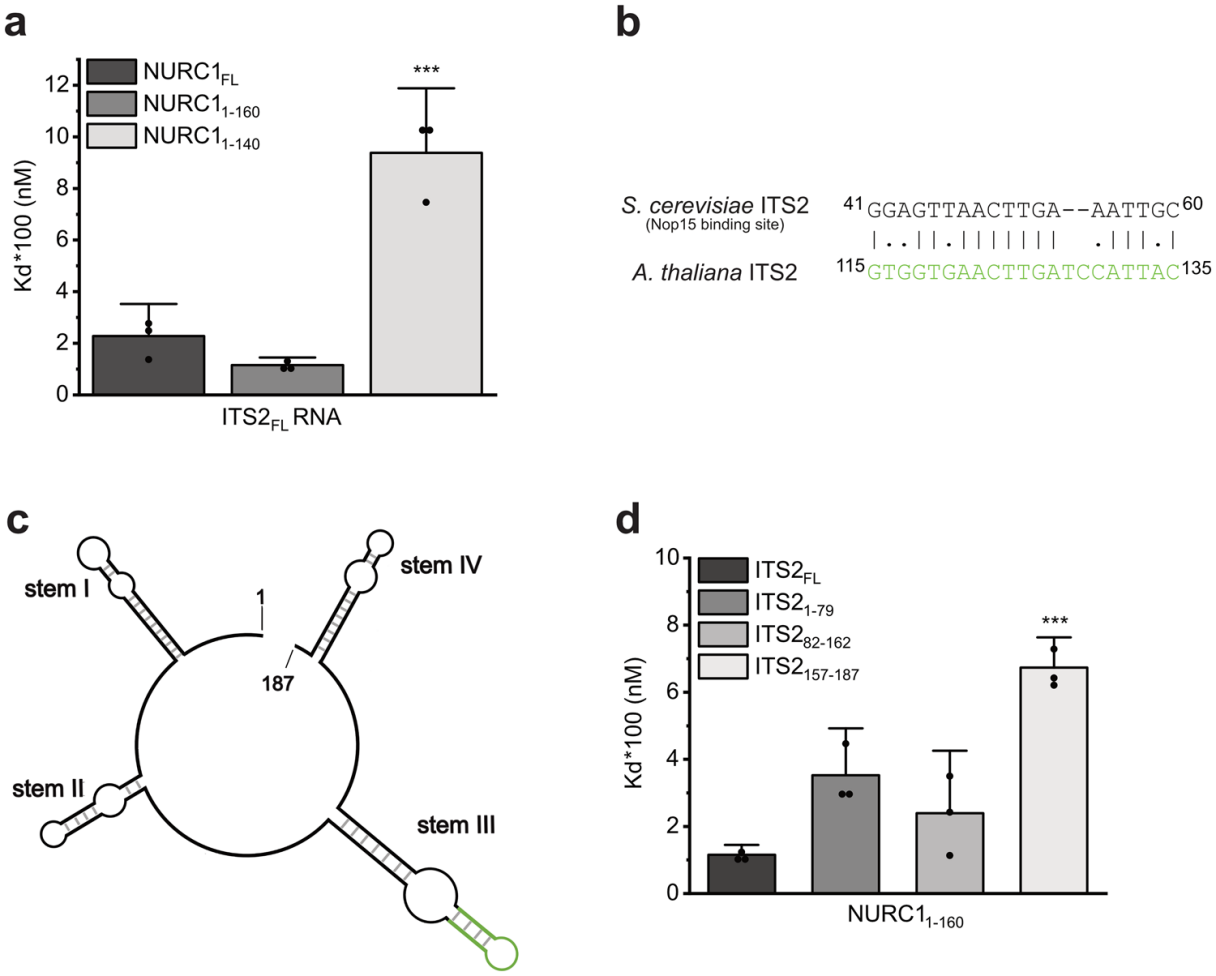
C-terminal truncation affects RNA binding and most ITS2 RNA stems bind equally to NURC1. (**a**) Kd values plot comparing the different truncated NURC1 versions. (**b**) Local alignment of Nop15 binding site and *A. thaliana* ITS2 using EMBOSS Water. (**c**) *A. thaliana* ITS2 rRNA. The green region of stem III shows 66.7% similarity with yeast’s ITS2 stem III.A. (**d**) NURC1_1-160_ binding affinities towards different ITS2 stems. Triplicates were measured and the results are plotted as mean ± standard deviation. The statistical analysis was done with one-way ANOVA (*p < 0.05, **p < 0.01 and ***p < 0.001) in OriginPro 2021b (OriginLab).

To test whether the RNA binding activity of yeast Nop15 is conserved in Arabidopsis NURC1, we studied binding of recombinant NURC1 to the internal transcribed sequence 2 (ITS2) RNA by MicroScale Thermophoresis (MST). For that purpose, we synthesized the ITS2 full-length RNA (ITS2 FL). Using MST, we were able to show that NURC1-FL and NURC1_1-160_ bind to ITS2 FL RNA with a Kd of 228 ± 83 nM and 116 ± 20 nM, respectively, both Kd values not being significantly different (Fig. 5a and Supplementary Table S5). Additionally, binding between NURC1_53-222_ and ITS2 FL RNA was detected (Supplementary Fig. S5a). However, fluorescence quenching at higher protein concentrations was observable. The SD-test was used to investigate whether the quenching resulted from protein aggregation or whether it was induced by ligand binding. No fluorescence recovery was observed after denaturation of NURC1_53-222_, thus indicating that quenching was a result of protein aggregation (Supplementary Fig. S5b). Consequently, the dissociation constant could not be determined for NURC1_53-222_. To screen for the actual binding sites of NURC1 in the Arabidopsis ITS2, we performed a local alignment analysis against the yeast stem III.A sequence by using EMBOSS Water (Fig. 5b). The stem-loop III end resulted in the best alignment (Fig. 5c). Accordingly, we synthesized three additional RNAs containing parts of ITS2, namely ITS2 1–79 (stem I and II), ITS2 82–162 (stem III), and ITS2 157–187 (stem IV). The secondary structure predictions of all synthesized ITS2 RNAs were given in Supplementary Fig. S6. The determined Kd values for binding of NURC1 FL and NURC1_1-160_ indicated considerable binding to ITS2 1–79 and ITS2 82–162, but less binding to ITS2 157–187 (Figs. 5d and S7). The removal of 20 additional amino acids in NURC1_1-140_ was sufficient to substantially reduce the binding affinity to ITS2 (Figs. 5a and S7, Supplementary Table S5), for example to a Kd = 938 ± 167 nM in the case of ITS2 FL.

### NURC1 possesses RNA chaperone-like activity to mediate RNA annealing

**Figure 6:**
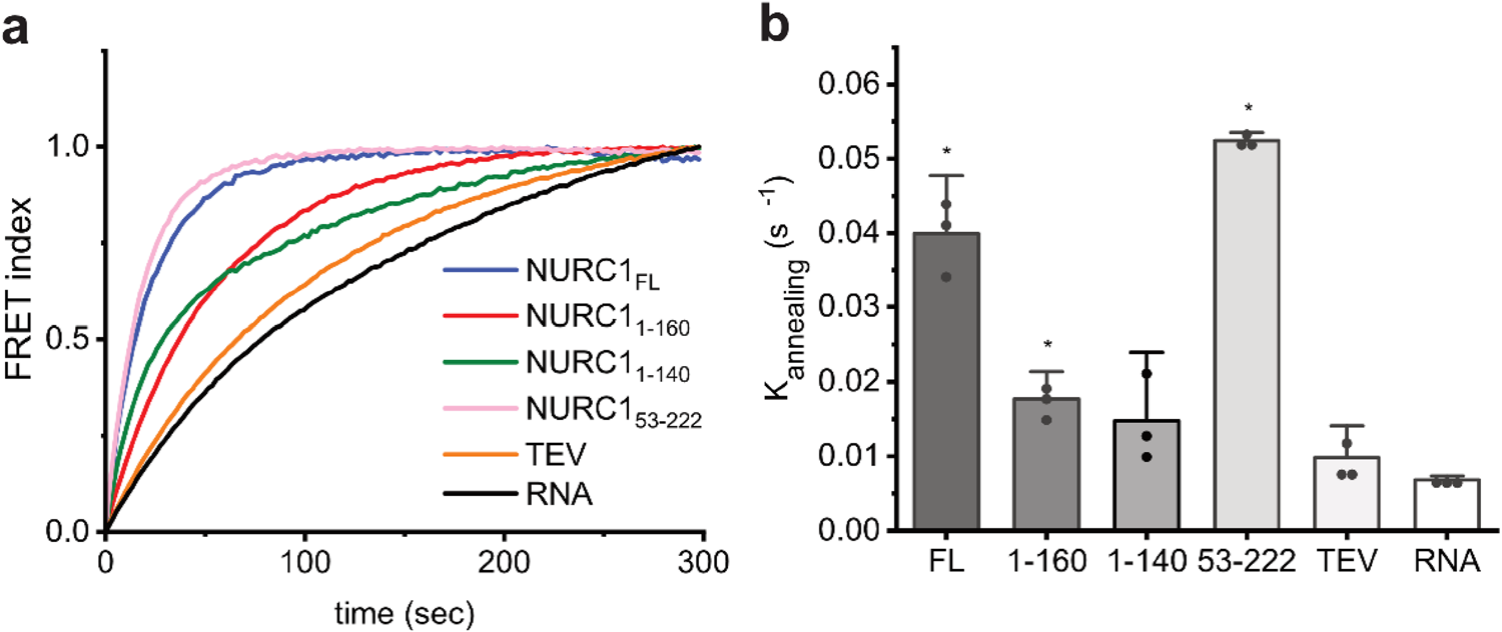
Both termini of NURC1 have an impact on its RNA chaperone-like activity. (**a**) Experimental curves were obtained by averaging triplicates and are shown as continuous lines that represent the changes of normalized FRET index (FCy5/FCy3) over time. The control sample RNA (black) consists only in the two 21-mer RNAs strands. A 1:5 (RNA:protein) molar ratio was used in all samples. The fluorescence was recorded for 300 s at 37 °C. (**b**) Overview of the K_annealing_ differences between samples. Triplicates were measured and the results are plotted as mean ± standard deviation. The statistical analysis was done by comparing each sample to the RNA control with two-sample t-test (*p < 0.05) in OriginPro 2021b (OriginLab).

In yeast, Nop15 has been shown to stabilize the ITS2 ring form by preventing the mispairing of stem III.A that may otherwise trap ITS2 in incorrect conformations and thus impair the assembly of pre-RNAs^26^. To test whether stabilization of the ITS2 ring form in Arabidopsis may be supported by NURC1, we investigated on its putative chaperone activity by using the real-time fluorescence resonance energy transfer (FRET) technique^31^. This experimental approach employed two single-stranded complementary 21-mer RNAs that were labelled with different fluorophores at their 5’-end, namely Cy5-21R^+^ and Cy3-21R^-^. Annealing of the two strands will result in energy transfer from Cy5 to Cy3 to decrease the fluorescence intensity of Cy5 (F_Cy5_) and to increase the fluorescence intensity of Cy3 (F_Cy3_). The normalized FRET index (F_Cy5_/F_Cy3_) was determined for a time of 300 s (Fig. 6a).

The TEV protease has about the same molecular weight as NURC1. When using TEV protease, the FRET index increased identically to the RNA control experiment, thus ruling out that annealing occurred due to a macromolecular crowding. In the presence of NURC1-FL, NURC1_1-160_, NURC1_1-140_, and NURC1_53-222_ the FRET index increased much faster than in the RNA control, indicating faster annealing, and reached a plateau after 300 s (Fig. 6a). The annealing constants K_annealing_ were determined through one-phase exponential association fitting to 0.0399 s^−1^ for NURC1 FL, 0.0177 s^−1^ for NURC1_1-160_ and 0.0525 s^−1^ for NURC1_53-222_, suggesting that these truncations had only a minor though significant effect on chaperone activity. The K_annealing_ was determined to 0.0148 s^−1^ for NURC1_1–140_, suggesting that the residues in between positions 140 and 160 do not affect the chaperone-like activity significantly (Fig. 6b, Table 1).

**Table 1:** NURC1 has RNA chaperone-like activity.

Protein	K_annealing_ (s^-1^)	Standard deviation	*p* value
NURC1_FL_	0.0399	5.19 × 10^–3^	3.86 × 10^–4^
NURC1_1-160_	0.0177	2.46 × 10^–3^	1.63 × 10^–3^
NURC1_1-140_	0.0148	6.13 × 10^–3^	8.81 × 10^–2^
NURC1_53-222_	0.0525	7.00 × 10^–4^	5.59 × 10^–8^
TEV	0.00978	2.85 × 10^–3^	1.50 × 10^–1^
RNA	0.00684	3.35 × 10^–4^	–

Annealing and displacement constants from the experimental data. *p* values were obtained by comparing each sample to the RNA control with a two-sample t-test.

## Discussion

The translation of mRNAs into proteins is essential for growth and development of organisms and requires biogenesis of functional ribosomes. While the assembly and function of ribosomes depends on ribosomal proteins (RPs), ribosome biogenesis also involves ribosome biogenesis factors (RBFs) for pre-rRNA processing into mature rRNAs. Many RPs and RBFs are capable of direct binding to RNA. We resolved the SAXS structure and showed RNA binding activity of the Arabidopsis protein NURC1, which is orthologous to the yeast RBF Nop15 that is crucial for cell growth in yeast^17^. The Nop15 ortholog from humans, NIFK, has a crucial function in cell cycle progression and may even promote cancer progression^37,38^, thus indicating an important role of Nop15-type proteins in development.

Our SEC-SAXS and modeling approaches revealed that the Arabidopsis NURC1 protein had an elongated shape (Fig. 3). Its N-terminus is likely highly disorganized and flexible and may have prevented successful crystallization of full-length NURC1, because crystallization of Nop15 has been achieved only with truncated N-terminus ^26^.

The RRM is typically around 90 amino acids long with a highly conserved architecture comprising four-stranded β-sheets packed against two α-helices, carrying a β_1_α_1_β_2_β_3_α_2_β_4_ fold. In many cases, proteins containing an RRM have been related to events in RNA biology, such as RNA processing, RNA editing, translation, and RNA degradation^39,40^. Commonly, the β-sheet moiety interacts with single-stranded RNA. The RNA-binding motifs RNP2 and RNP1 are located in β1- and β3-sheets, respectively. Sequence and structure alignments of NURC1 with its orthologs, Nop15 and NIFK, confirmed the presence of a *bona fide* RRM in NURC1, with conserved aromatic residues F105 and F108 in RNP1 and Y63 in RNP2 for RNA binding (Fig. 4). RNA binding is usually achieved by stacking interactions between the sidechain of aromatic residues and the cognate nucleotides^40,41^.

The presented structural model predicted the presence of an additional α-helical region in the C-terminus of NURC1, as has been described for the yeast Nop15 protein^26^. Apparently, NURC1 resembled the structural features of Nop15.

In line with the presence of the RRM in the NURC1 structure, we demonstrated that indeed NURC1 was able to bind to the internal transcribed sequence 2 (ITS2) RNA contained in the Arabidopsis polycistronic pre-rRNA (Figs. 5a and S6, Supplementary Table S5). Yeast Nop15 has been shown to bind to the stem III.A sequence of yeast ITS2. In line with that, NURC1 exhibited high binding affinity to stem III, but also to stem I and II of the Arabidopsis ITS2 sequence. For both, NURC1 and Nop15 binding affinity diminished upon deletion of the C-terminus, suggesting a contribution of the C-terminus to RNA binding (Figs. 5a and S6, Supplementary Table S5)^26^. In contrast to Nop15, however, this did not correlate with the α-helical region in the C-terminus of NURC1, because the affinity was similar in NURC1 and NURC1_1-160_, in which the C-terminal α-helices were removed. The affinity decreased only after removal of 20 additional amino acids in NURC1_1-140_. It has been shown in the Nop15 crystal structure that the C-terminus binds to amino acid residues in the RRM that are important for RNA binding. Rearrangement of the C-terminus may be important for the putative function of Nop15 in stabilizing the III.A stem loop^25,26^. Since processing at the ITS2 involves dynamic conformational changes between its ring and its hairpin structure^27^, it has been speculated that Nop15 may stabilize the ITS2 ring structure prior to hairpin formation to prevent misfolding at this site^26^. Although the detailed high-resolution structure of NURC1 has to be elucidated in the future, the chaperone-like function of NURC1 that allowed for annealing of two RNA sequences may support a similar function of NURC1 in Arabidopsis (Fig. 6). The determined chaperone-like activities were in the expected range^31^. In addition, we could show that C-terminal deletion in NURC1_1-160_ and NURC1_1-140_ resulted in less efficient annealing of RNA when compared to the RNA annealing activity of full-length NURC1. In contrast, N-terminal deletion in NURC1_53-222_ resulted in an increase of RNA annealing efficiency. In contrast to the pure NURC1_53-222_ protein that did not exhibit aggregation as demonstrated by our SEC-MALLS data, NURC1_53-222_ aggregated in the presence of RNA (Supplementary Fig. S5). Thus, the RNA may be dragged into close proximity within NURC1_53-222_ aggregates and may lead to an increased FRET signal due to localized crowding.

The role of yeast Nop15 and its human ortholog NIFK in pre-rRNA processing and thus rRNA maturation has been described, but the underlying mechanism may be slightly different^17,23,38,42^. Though the actual biological function of NURC1 has to be unraveled in future investigations, the role of other Arabidopsis orthologs of the nucleolar Nop15 pre-60S complex has previously been studied. For example, the nucleolar Nug1 ortholog NUCLEOSTEMIN1 (NSN1) is a putative GTPase that binds to 25S and 18S rRNA^21^. In Arabidopsis *nsn1* mutants lacking the expression of *NSN1*, 25S rRNA maturation and biogenesis of the 60S ribosome subunit were delayed, resulting in severe developmental phenotypes^21,22,43^. The Arabidopsis PESCADILLO protein AtPES, which is an ortholog of Nop7 in yeast, interacts with NSN1^21^. Depletion of AtPES also resulted in delayed maturation of 25S rRNA and in defective biogenesis of 60S subunits, thus leading to severe developmental defects in Arabidopsis^44^. The Rat1 ortholog XRN2 was shown to be crucial for primary cleavage in the pre-rRNA^13^. Two orthologs of the yeast Brx1 protein with redundant function in pre-rRNA processing steps have been identified^12^. SMALL ORGAN4 (SMO4) is the most likely ortholog of yeast Nop53 and shares the participation in 5.8S maturation with the yeast component of the pre-60S complex. Loss of function of SMO4 resulted in increased levels of 5.8S and 18S precursors and in a changed ratio between 40 and 60S ribosome subunits^45^. These examples suggest the conservation of functions of the yeast Nop15 pre-60S complex in Arabidopsis orthologs. It will be interesting to find out whether NURC1 binding to ITS2 and its function in annealing of RNAs also point to a function in pre-rRNA processing, ribosome biogenesis, and thus plant development.

## Supplementary Information

Supplementary Information.

## Data Availability

Materials described in the manuscript will be freely available to any researcher wishing to use them for non-commercial purposes. Request should be directed to the corresponding author. The SAXS data for NURC1 have been deposited to the Small Angle Scattering Biological Data Bank (SASBDB) (https://www.sasbdb.org/data/SASDRN5/grptc5azg8).
