# Supplementary material for: Structural and functional analysis of a plant nucleolar RNA chaperone-like protein
Source: Sci Rep. 2023 Jun 14;13:9656. doi: 10.1038/s41598-023-36426-4 (PMC10267116; doi:10.1038/s41598-023-36426-4)
Supplement: Supplementary file 1 — Supplementary Information. [file 41598_2023_36426_MOESM1_ESM.pdf]

Structural and Functional Analysis of a Plant Nucleolar RNA Chaperone-Like Protein

Rita Fernandes[1,2], Anna Ostendorp[2], Steffen Ostendorp[2], Judith Mehrmann[1], Sven Falke[3], Melissa Ann Graewert[4], Magdalena Weingartner[1], Julia Kehr[2], Stefan Hoth[1,*]

[1] Molecular Plant Physiology, Institute of Plant Science and Microbiology, Department of Biology, Universität Hamburg, Hamburg, Germany

[2] Molecular Plant Genetics, Institute of Plant Science and Microbiology, Department of Biology, Universität Hamburg, Hamburg, Germany

[3] Center for Free-Electron Laser Science (CFEL), Deutsches Elektronen Synchrotron (DESY), Notkestrasse 85, 22607 Hamburg, Germany

[4] European Molecular Biology Laboratory (EMBL) Hamburg Unit, Hamburg, Germany

[*]Corresponding author: Stefan Hoth; E-mail: stefan.hoth@uni-hamburg.de

Supplementary Figure S1

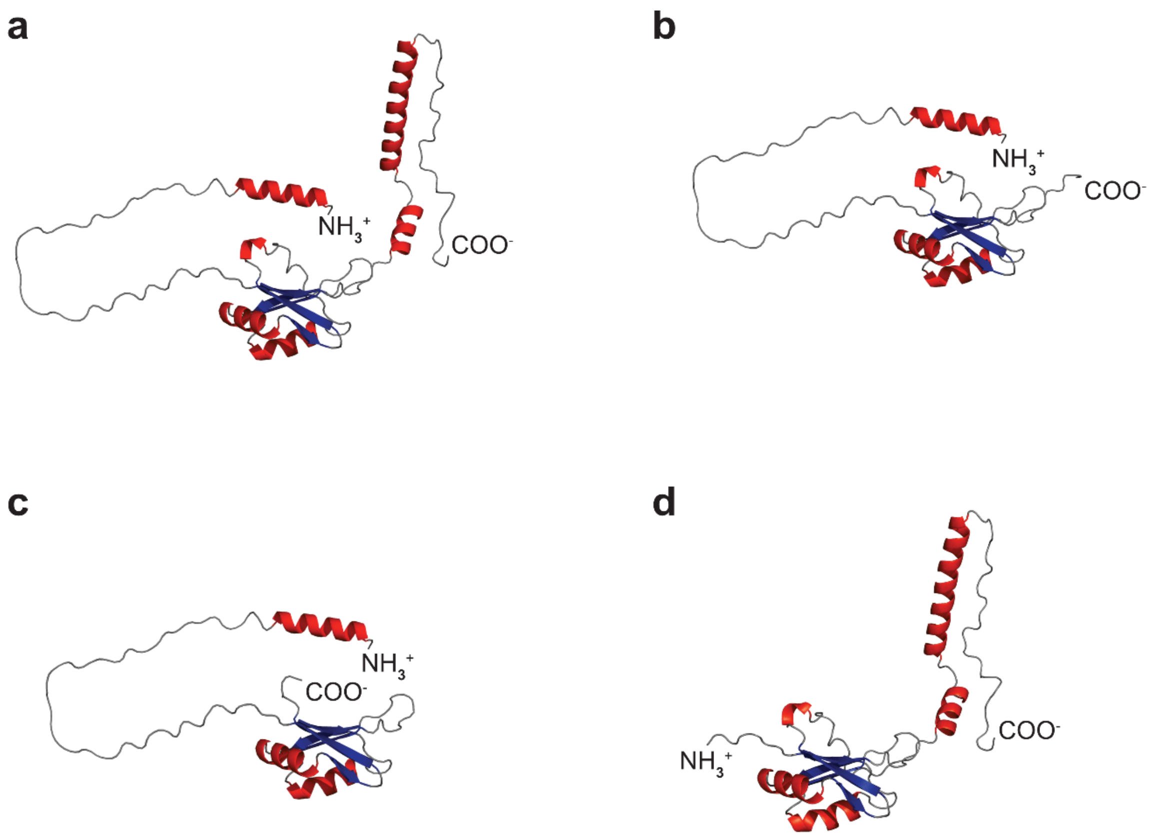

Figure S1. Representation of the predicted secondary structure of NURC1. (**a**) NURC1 full length. (**b**) NURC1$_{1\text{-}160}$. (**c**) NURC1$_{1\text{-}140}$ (**d**) NURC1$_{53\text{-}222}$. The full-length structure is the AlphaFold model Q9LZ65 and the truncations were achieved by removing the residues to the desired position. Both termini are represented as expected at neutral pH.

Supplementary Figure S2

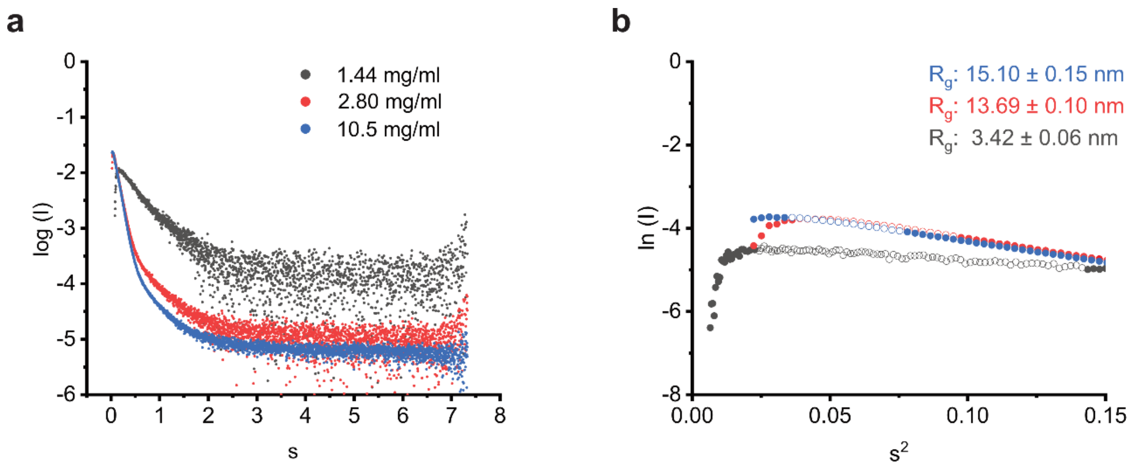

Figure S2. SAXS analysis of NURC1$_{FL}$ in batch mode. (**a**) Scattering curves determined at three different concentrations. (**b**) Guinier fit (open spheres) of data at the respective concentrations. The resulting R_g values are shown as insets and a linear fit was applied to them resulting in a Pearson's R value of 0.7.

Supplementary Figure S3

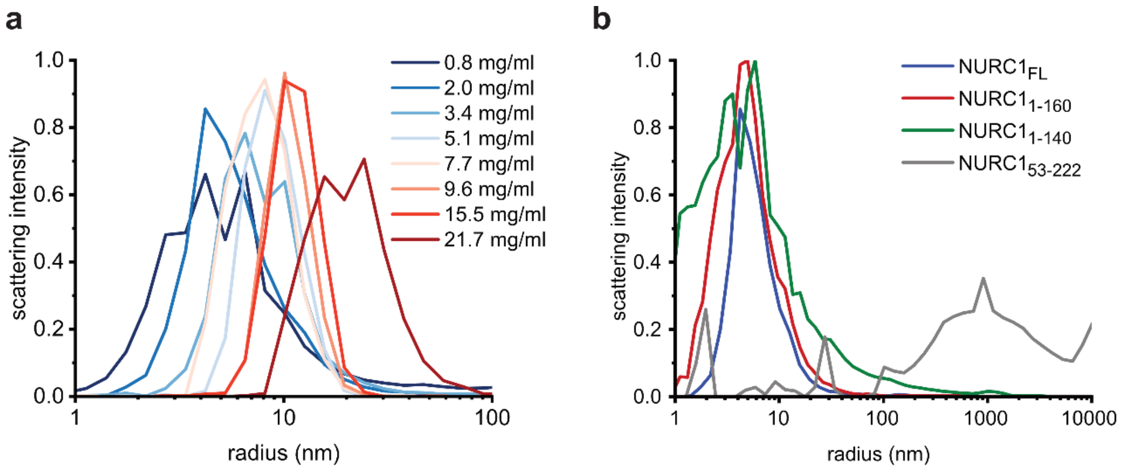

Figure S3. DLS scattering profiles of NURC1 revealed dependency of polydispersity on concentration and C-terminal truncation. (**a**) NURC1$_{FL}$ at different concentrations. (**b**) NURC1$_{FL}$ (2 mg/ml) in blue, NURC1$_{1\text{-}160}$ (2.4 mg/ml) in red, NURC1$_{1\text{-}140}$ (1.0 mg/ml) in green, and NURC1$_{53\text{-}222}$ (2.4 mg/ml) in grey. All samples were centrifuged at 20 000 x g for 10 min. The concentrations were measured by using a NanoDrop spectrophotometer, and the molar extinction coefficients were determined by using the ProtParam tool, assuming all cysteines to be reduced.

Supplementary Figure S4

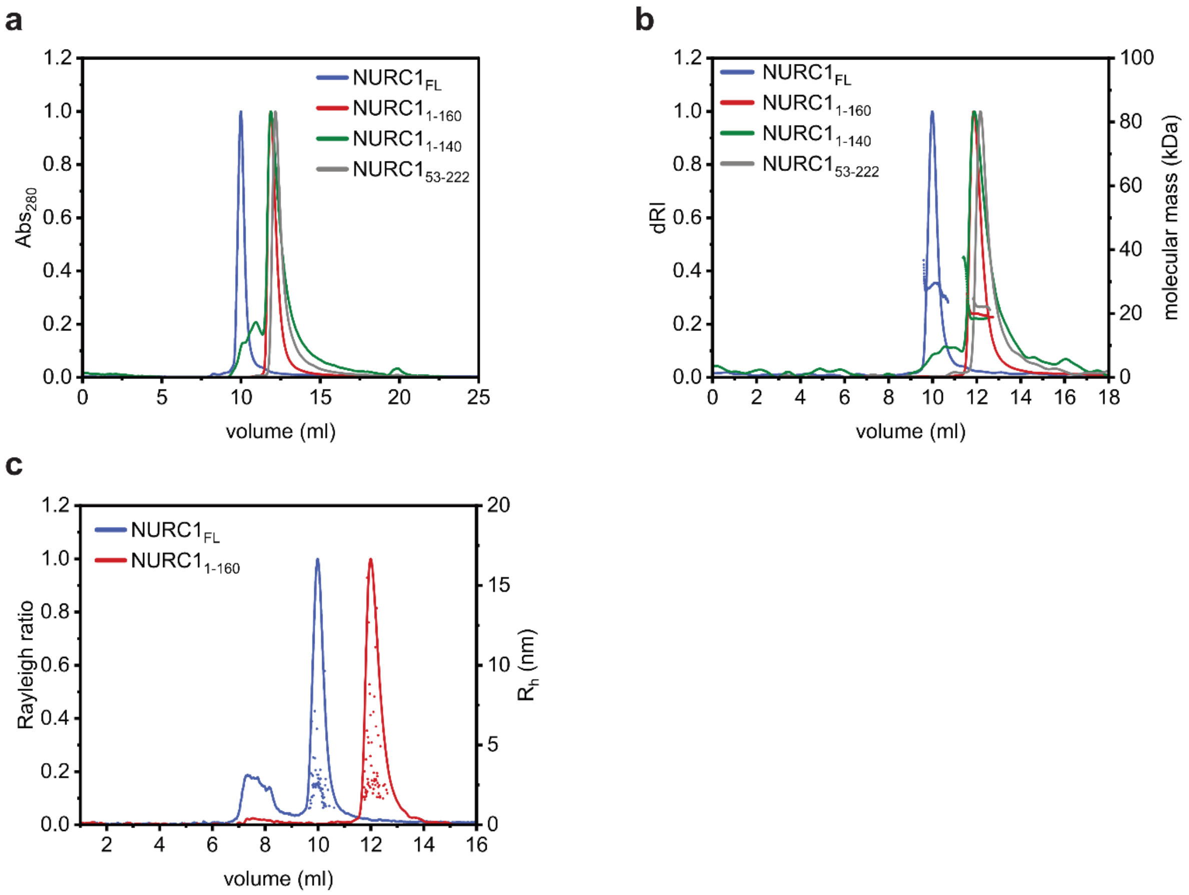

Figure S4. SEC-MALLS of NURC1 (**a**) SEC profiles of full-length and truncated NURC1 proteins. (**b**) Differential refractive index vs volume plot showing the elution peaks for the full-length and truncated NURC1 proteins (full line) and respective molar mass predictions (spheres). (**c**) Rayleigh ratio vs volume plot showing the elution peaks of NURC1$_{FL}$ and NURC1$_{1\text{-}160}$ (full line) and the R_h prediction (spheres).

Supplementary Figure S5

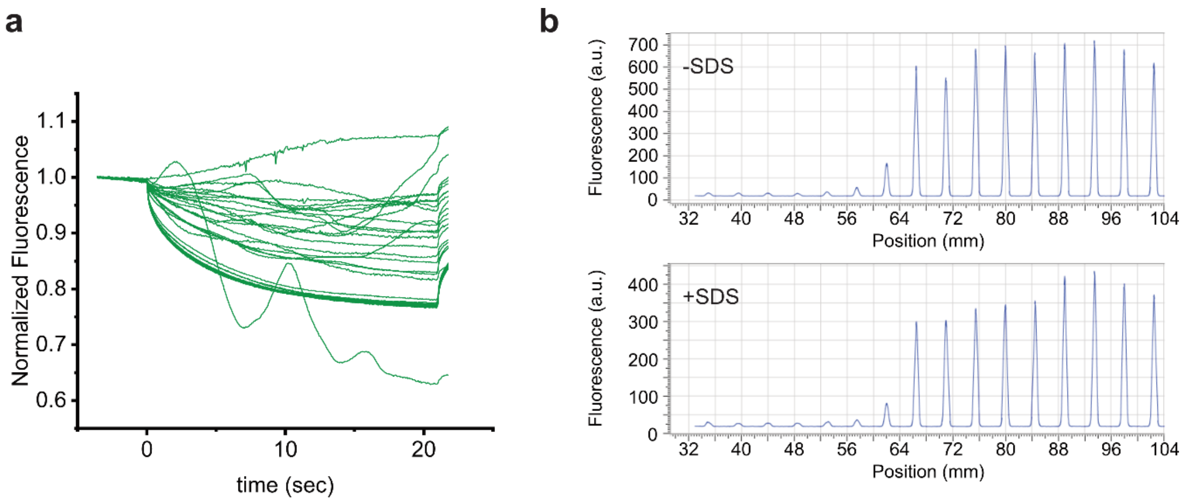

Figure S5. NURC1$_{53\text{-}222}$ is aggregated at higher concentrations. (**a**) MST traces (green lines) of NURC1$_{53\text{-}222}$ (the concentration increases from the bottom to top) and cy5 labelled ITS2$_{FL}$ (20 nM). (**b**) SD-test of NURC1$_{53\text{-}222}$. The protein concentration decreases from the left to the right. Capillary scan of samples without (top) and with (bottom) SDS treatment.

Supplementary Figure S6

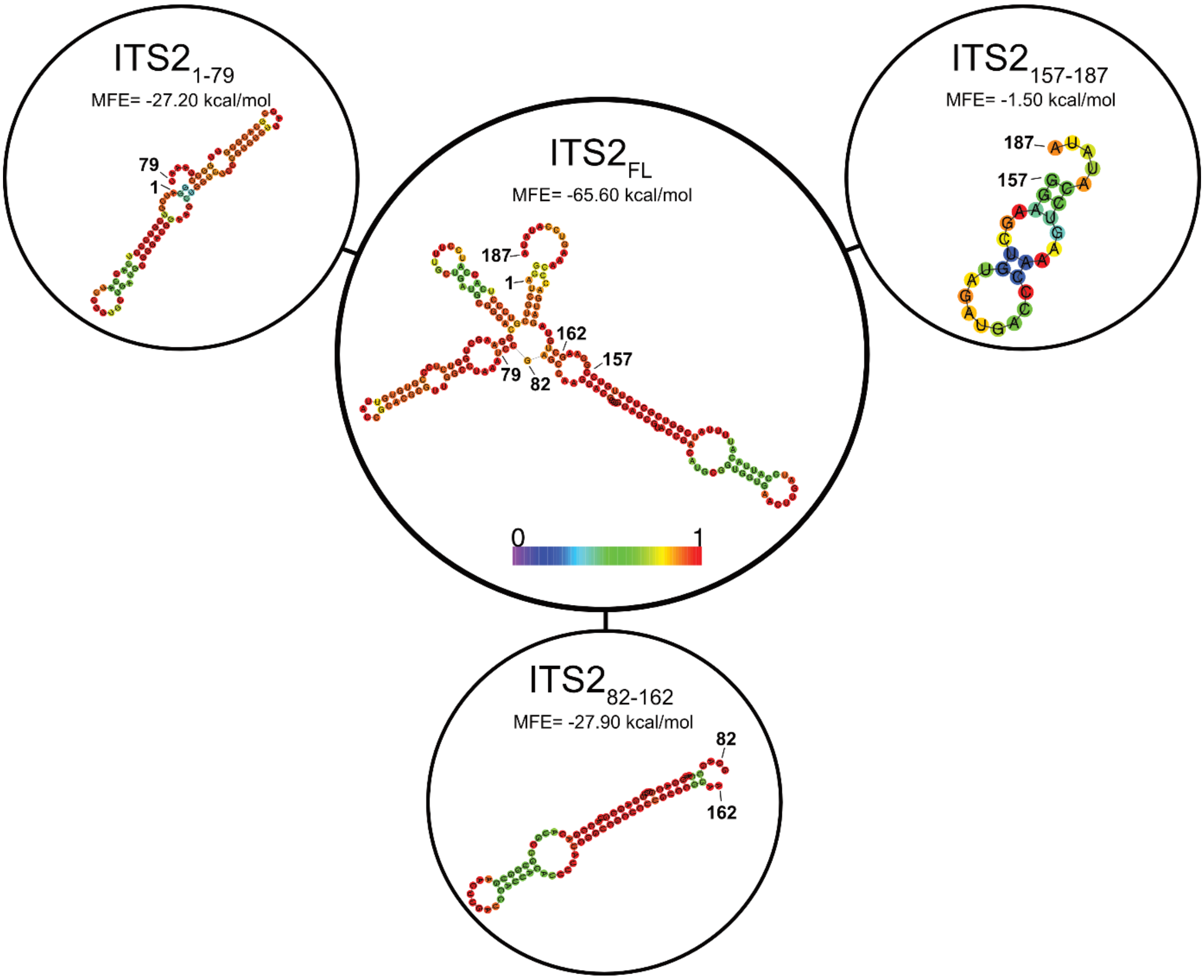

Figure S6. RNAfold secondary structure prediction of ITS2 RNA fragments. Minimum free energy (MFE) structures of each fragment are represented. Base pair probabilities are shown in colours. When not paired the colour represents the probability of not being paired. The colours range from blue (0) to red (1). Structures were generated by using the Vienna RNA Websuite (Gruber *et al.*, *Nucleic Acids Research*, Vol. 36, 2008).

Supplementary Figure S7

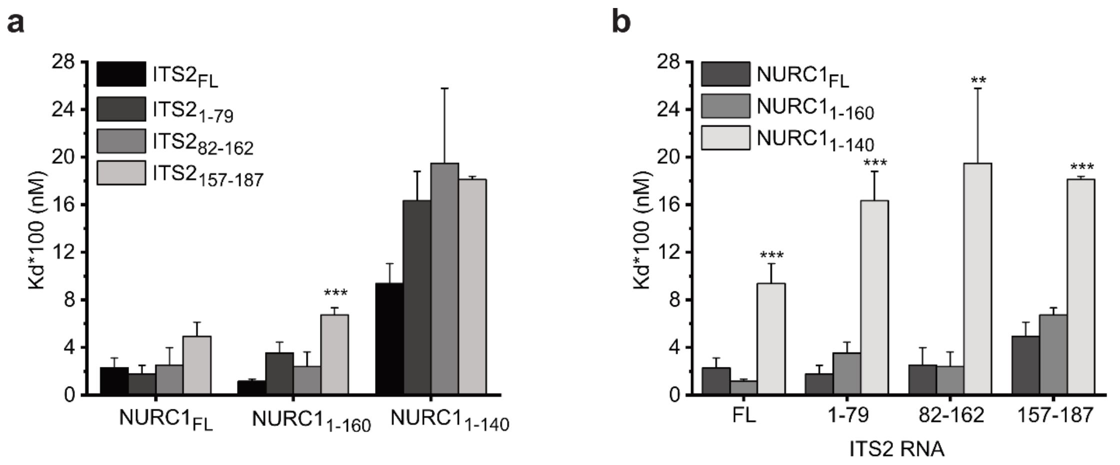

Figure S7. Comparison of dissociation constants determined by MST. (**a**) Plot of the determined Kd values comparing the binding between different truncated NURC1 proteins and ITS2 RNA fragments. (**b**) $NURC1_{FL}$, $NURC1_{1\text{-}160}$ and $NURC1_{1\text{-}140}$ binding affinities towards different ITS2 regions. Triplicates were measured and the results were plotted as mean ± standard deviation. The statistical analysis was done with one-way ANOVA (*p <0.05, **p <0.01 and ***p <0.001) in OriginPro 2021b (OriginLab).

Supplementary Table 1

Table S1. List of primers used for cloning and *in vitro* transcription.

ID	Sequence 5´ to 3´
At5g04600_FRET_fw	GGGGACAAGTTTGTA CAAAAAAGCAGGCTCCACCATGGGTGCCAAGGCGAAG
At5g04600_FRET_rev	GGGGACCACTTTGTA CAAGAAAGCTGGGTTGTCTTCTTCACTAAACT
At5g05210_FRET_fw	GGGGACAAGTTTGTA CAAAAAAGCAGGCTCCACCATGACTAAAACTCGTG
At5g05210_FRET_rev	GGGGACCACTTTGTA CAAGAAAGCTGGGTTGTTTCCACCTTCATTG
At3g22660_FRET_fw	GGGGACAAGTTTGTA CAAAAAAGCAGGCTCCACCATGTCATTGGAAGAGGAT
At3g22660_FRET_rev	GGGGACCACTTTGTA CAAGAAAGCTGGGTTCCTCTTCTGCCTCTTG
MAIL1_FRET_fw	GGGGACAAGTTTGTA CAAAAAAGCAGGCTCCACCATGGATATTGATGTC
MAIL1_FRET_rev	GGGGACCACTTTGTA CAAGAAAGCTGGGTTCGAGTCTTCCTTTTG
NURC1$_{FL}$_NdeI_fw	GATCCATATGGGGTGCCAAGGCGAAG
NURC1$_{FL}$_XhoI_rev	GATCCTCGAGGTCTTCTTCACTAAACTTGATCCTC
NURC1$_{1\text{-}160}$_fw	CAAACCAGTGGATTCGGTTCAGTAAGAGCGAAGACAACTTAATAAGG
NURC1$_{1\text{-}160}$_rev	CCTTATTAAGTTGTCTTCGCTCTTACTGAACCGAATCCACTGGTTTG
NURC1$_{1\text{-}140}$_fw	GCTTAAAGTCCATGTCATTGAACCAGAGAATTAAAAACCCAATCTGTGGAG
NURC1$_{1\text{-}140}$_rev	CTCCACAGATTGGGTTTTTAATTCTCTGGTTCAATGACATGGACTTTAAGC
NURC1$_{53\text{–}222}$_fw	TTAAGGTCTCGTTGCCCACCTCTTCAGAATAAAGCTACTGTGCTATAC
NURC1$_{53\text{-}222}$_rev	TTAAGGTCTCGAAGCTTAGTCTTCTTCACTAAACTTGATCCTCTTCGGG
45SrDNA_HindIII_fw	GACTGCAGAGGCCTGCATGCATAATACGACTCACTATAGGGAATTCCTAGTAAGCGCGAGT CATCAGC
45SrDNA_HindIII_rev	ACCATGATTACGCCACTAGCATAACCCCTTGGGGCCTCTAAACGGGTCTTGAGGGGTTTTTT GGAATTCGTTAAGGAGCTGTTGCTTTGTTAGTG
T7p_ITS2$_{FL}$_fw	GAAATTAATACGACTCACTATAGGATCGTCGTCCCTCACCATCCTT
ITS2$_{FL}$_rev	GCCTTCGACATCTACTGGGTTTCAGGTATAT
T7p_ITS2$_{1\text{-}79}$_fw	GAAATTAATACGACTCACTATAGGATCGTCGTCCCTCACCATCCTTTGCTGATGCGGGACGG AAGCTGGTCTCCCGTGTGTTACCGCACGCGTTGGCCTAATT
ITS2$_{1\text{-}79}$_rev	AATTAGGCCAACGCGTGCGGTAACACACGGGAGACCAGCTTCCGTCCCGCATCAGCAAAG GATGGTGAGGGACGACGATCCTATAGTGAGTCGTATTAATTTC
T7p_ITS2$_{82\text{-}162}$_fw	GAAATTAATACGACTCACTATAGGAGCCAAGGACGCCTGGAGCGTACCGACATGCGGTGGT GAACTTGATCCATTACATTTTATCGGTCGCTCTTGTCCGGAA
ITS2$_{82\text{-}162}$_rev	TTCCGGACAAGAGCGACCGATAAAATGTAATGGATCAAGTTCACCACCGCATGTCGGTACGC TCCAGGCGTCCTTGGCTCCTATAGTGAGTCGTATTAATTTC
T7p_ITS2$_{153\text{-}187}$_fw	GAAATTAATACGACTCACTATAGGAAGCTGTAGATGACCCAAAGTCCATATA
ITS2$_{153\text{-}187}$_rev	TATATGGACTTTGGGTCATCTACAGCTTCCTATAGTGAGTCGTATTAATTTC

Supplementary Table 2

Table S2. Sample information and SEC-SAXS parameters.

**Sample information**	Organism	*A. thaliana*
	Source	In house purification
	UniProt ID	Q9LZ65
	Calculated Extinction Coefficient [A280, 0.1%(w/v)]	0.459
	"v" ¯from chemical composition (cm3.g-1)	0.743
	Particle contrast from sequence and solvent constituent	2.727 (12.267-9.540)
	M from chemical composition (Da)	25262
**SEC parameters**	SEC column	Superdex 10/300 G
	Loading concentration	18.0 mg/ml
	Injection volume	87µl
	Flow rate	0.5 ml/min
	Solvent	50 mM HNa_2PO_4 pH 7.5, 300 mM NaCl, 5% glycerol, 1 mM DTT
**SAXS parameters**	X-ray source	PETRA III (DESY, Hamburg, Germany), Beamline P12
	Detector	Photon counting Pilatus 6M (423.6 x 434.6 mm^2)
	Wavelength (Å)	1.24
	Detector distance (m)	3
	Focal spot (mm)	0.2 x 0.12
	Exposure time (sec)	Batch: 0.045<br>SEC-SAXS: 0.995
	Sample temperature (°C)	20

Supplementary Table 3

Table S3. SEC-MALLS parameters and experimental results.

Sample	$C_{loading}$(mg/ml)	$V_{loading}$ (µl)	$V_{elution}$ (ml)	$Expected_{MW}$ (kDa)	$Experimental_{MW}$ (kDa)	R_h (nm)
$NURC1_{FL}$	18.00	10	9.99	25.26	27.65	2.4
$NURC1_{1\text{-}160}$	17.40	20	11.87	17.76	19.94	2.4
$NURC1_{1\text{-}140}$	2.33	40	11.89	15.42	18.45	-
$NURC1_{53\text{-}222}$	2.37	40	12.17	19.92	22.06	-

Supplementary Table 4

Table S4. *Ab initio* (DAMMIN and GASBOR) and CRYSOL fitting χ^2 results for the different models.

Model	ID	χ^2
DAMMIN P1	-	1.06
GASBOR P1	-	1.56
AlphaFold	Q9LZ65 (UniProt)	11.97
i-Tasser	-	18.72
Nop15 CryoEM	3JCT, Chain OA (PDB)	55.57
Nop15 crystal - monomer	5T9P (PDB)	253.22

Supplementary Table 5

Table S5. Summary of the binding affinities between different NURC1 protein versions and RNA regions.

				Protein comparison		*RNA comparison*	
**RNA**	**Protein**	**Kd (nM)**	**SD**	**p-value**	**Fold change**	**p-value**	**Fold change**
$ITS2_{FL}$	$NURC1_{FL}$	228	83	-	1.0	-	1.0
	$NURC1_{1\text{-}160}$	116	20	4.6×10^{-1}	0.5	-	1.0
	$NURC1_{1\text{-}140}$	938	167	4.8×10^{-4}	4.1	-	1.0
$ITS2_{1\text{-}79}$	$NURC1_{FL}$	177	73	-	1.0	9.7×10^{-1}	0.8
	$NURC1_{1\text{-}160}$	353	93	4.3×10^{-1}	2.0	7.6×10^{-2}	3.0
	$NURC1_{1\text{-}140}$	1634	246	7.1×10^{-5}	9.2	2.5×10^{-1}	1.7
$ITS2_{82\text{-}162}$	$NURC1_{FL}$	251	147	-	1.0	10×10^{-1}	1.1
	$NURC1_{1\text{-}160}$	239	124	10×10^{-1}	0.9	4.9×10^{-1}	2.1
	$NURC1_{1\text{-}140}$	1948	631	3.8×10^{-3}	7.8	7.0×10^{-2}	2.1
$ITS2_{157\text{-}187}$	$NURC1_{FL}$	494	119	-	1.0	5.2×10^{-2}	2.2
	$NURC1_{1\text{-}160}$	673	60	7.0×10^{-2}	1.4	7.9×10^{-4}	5.8
	$NURC1_{1\text{-}140}$	1812	27	2.1×10^{-6}	3.7	1.2×10^{-1}	1.9
